# Variation in accessibility of the population to an Emergency Medical Communication Centre: a multicentre observational study

**DOI:** 10.1186/s13049-019-0667-6

**Published:** 2019-10-28

**Authors:** Yann Penverne, Brice Leclere, Eric Lecarpentier, Jean-Sébastien Marx, Benjamin Gicquel, Laurent Goix, Paul-Georges Reuter

**Affiliations:** 1 Samu 44, Department of Emergency Medicine, University Hospital of Nantes, Nantes, France; 2 Department of Medical Evaluation and Epidemiology, Nantes University Hospital, Nantes, France; 3 SAMU 94, Assistance Publique-Hôpitaux de Paris, Hôpitaux Universitaires Henri Mondor, F-94000 Creteil, France; 4 SAMU 75, Assistance Publique-Hôpitaux de Paris, Hôpital Universitaire Necker-Enfants-Malades, 75730 Paris, France; 5 SAMU 85, Centre hospitalier départemental Vendée, La Roche Sur Yon, France; 6 SAMU 93, Assistance Publique-Hôpitaux de Paris, Hôpitaux Universitaires de Paris-Seine-Saint-Denis, Hôpital Avicenne, 93009 Bobigny, France; 7 SAMU 92, Assistance Publique-Hôpitaux de Paris, Hôpital Raymond Poincaré, 104, Boulevard Raymond Poincaré, 92380 Garches, France; 8 UMR 1027, Université Paul Sabatier Toulouse III, Inserm, Toulouse, France

**Keywords:** Emergency Medical Communication Centre, Scheduling process, Work force management, Accessibility

## Abstract

**Background:**

Access to an Emergency Medical Communication Centre is essential for the population in emergency situations. Handling inbound calls without delay requires managing activity, process and outcome measures of the Emergency Medical Communication Centre to improve the workforce management and the level of service. France is facing political decisions on the evolution of the organisation of Emergency Medical Communication Centres to improve accessibility for the population. First, we aim to describe the variation in activity between Emergency Medical Communication Centres, and second, to explore the correlation between process measures and outcome measures.

**Methods:**

Using telephone activity data extraction, we conducted an observational multicentre study of six French Emergency Medical Communication Centres from 1 July 2016 to 30 June 2017. We described the activity (number of incoming calls, call rate per 1000 inhabitants), process measure (agent occupation rate), and outcome measure (number of calls answered within 20 s) by hourly range and estimated the correlation between them according to the structural equation methods.

**Results:**

A total of 52,542 h of activity were analysed, during which 2,544,254 calls were received. The annual Emergency Medical Communication Centre call rate was 285.5 [95% CI: 285.2–285.8] per 1000 inhabitants. The average hourly number of calls ranged from 29 to 61 and the call-handled rate from 75 to 98%. There are variations in activity between Emergency Medical Communication Centres. The mean agent occupation rate was correlated with the quality of service at 20 s (coefficient at − 0.54). The number of incoming calls per agent was correlated with the mean occupation rate (coefficient at 0.67). Correlation coefficients varied according to the centres and existed between different process measures.

**Conclusions:**

The activity dynamics of the six Emergency Medical Communication Centres are not identical. This variability, illustrating the particularity of each centre, must be accurately assessed and should be taken into account in managerial considerations. The call taker occupation rate is the leverage in the workforce management to improve the population accessibility.

## Background

The Emergency Medical Communication Centre (EMCC) serves as the first point of contact between the caller and the emergency services. Therefore, it is vital that these call centres operate quickly and provide good quality of service. For cardiac arrest, for example, it has been proven that EMCCs save lives by promoting an appropriate prioritisation and response among all incoming calls and by providing potential life-saving guidance, advice or instructions [1–6]. A recent systematic review highlighted the poor level of evidence regarding the medical dispatching systems’ accuracy [7]. Another systematic review focussed on interventional studies performed in an EMCC. Few studies were included, and the majority had a poor level of quality [8]. Authors of both systematic reviews reported the need to improve research in this field [7, 8].

Fast medical care is only possible if emergency calls are immediately taken and appropriate operational resources are promptly dispatched. This requires a sufficient number of call takers to handle incoming calls and assess and prioritise requests [9]. Determining such a staffing level involves a detailed analysis of the examined EMCC’s processes in order to reach an efficient organisation. Activity and process measures (Table 1) are used to assess quantitative performance and an EMCC’s accessibility. In this field the literature illustrates a lack of knowledge. The guidelines for handling emergency calls vary from country to country, with differences in performance thresholds, but they all underline the common objective of reducing response time [10–12].

**Table 1:** Activity, process measures and outcome definition

	Definitions
Activity measures	
Number of calls	Number of incoming calls
Call rate per 1000 inhabitants	Number of incoming calls per 1000 inhabitants
Connected call taker	Connection status to the advanced telephony system. A connected call taker could be busy on a call or available to handle an incoming call
Process measures	
Occupation rate (OR)	Proportion of time when call takers are on live calls or completing work associated with the calls
Average call duration (ACD)	Average time on call for a call taker
Outcome	
Calls answered in 20s (QS20)	Rate of answered calls within 20 s

France is facing political decisions on the evolution of the organisation of Emergency Medical Communication Centres to improve accessibility for the population. Current French health policy highlights the challenge of a national emergency quality response including the EMCC operational management. To the best of our knowledge, no nationwide multicentre evaluation of EMCCs has been carried out. The homogenisation of an operational management, based on a potential homogeneity between the centres, is therefore theoretical. In order to provide input for operational guidelines on population accessibility, we aimed (1) to analyse the variation in activity between EMCCs and (2) to explore the correlation between process measures and outcome measures at the multicentre level.

## Methods

### Study design and setting

We conducted a multicentre observational study using structural equation models (SEM), an original method [13]. These types of models are useful to explore the relationship between process indicators and their correlation to outcome measures. They are commonly used in social and environmental epidemiology to deal with complex causal pathways and highly correlated data.

France is administratively divided into 100 departments, 96 of which are in metropolitan France. Every department has its own EMCC, called “Service d’Aide Médicale Urgente”, (SAMU) that provides the response to both urgent and non-urgent medical calls [14]. These calls are first handled by call takers and are transferred, depending on the severity of the patient’s condition, to a general practitioner or an emergency physician. In response to a call, physicians can give medical advice, recommend going to a medical care facility, or send first aid responders or a mobile intensive care unit (emergency medical system with an emergency physician on board).

Our study was based on a convenience sample of six French EMCCs chosen for their ability to provide harmonised activity data and using data collected prospectively from 1 July 2016 to 30 June 2017. No system changes occurred during this period. These centres covered about 9 million people, i.e., 14% of the population of metropolitan France (Table 2). In the rest of the article, the centres will be referred to anonymously as centre 1 to centre 6.

**Table 2:** General characteristics, activity and process measures of each centre from 1 July 2016 to 30 June 2017

	Overall	Centre 1	Centre 2	Centre 3	Centre 4	Centre 5	Centre 6
Incoming calls	2,544,254	431,676	532,518	422,710	521,807	380,930	254,613
Population size	8,910,900	1,413,300	2,168,500	1,601,100	1,646,100	1,401,200	680,200
Area covered		Mixed	Urban	Urban	Urban	Mixed	Mixed
EMCC calls handled rate(per 1000 inhabitants; 95% CI)	285.5 [285.2–285.8]	305.4 [304.7–306.2]	245.6 [245.0–246.1]	263.9 [263.2–264.6]	317.0 [316.3–317.7]	271.9 [271.1–272.6]	374.3 [373.2–375.5]
Connected call takers	4.9 ± 1.5	4.7 ± 1.7	4.7 ± 1.1	4.7 ± 0.9	5.4 ± 1	3.9 ± 1.1	6 ± 1.7
Incoming calls per hour	48.4 ± 27	49.3 ± 24.3	61.0 ± 33.5	48.4 ± 23.3	59.8 ± 25.8	43.6 ± 19.9	29.1 ± 18.8
Handled calls per hour	43.3 ± 21.7	48.2 ± 23.2	45.6 ± 20.8	45.2 ± 21	53.6 ± 21.4	39.5 ± 16.9	28.4 ± 17.5
Average call duration (sec)	75.5 ± 32.0	101.7 ± 19.4	70.7 ± 12.6	52.5 ± 10.2	50.9 ± 7.9	52.6 ± 9.4	124.5 ± 24.3
Calls per operator	10.1 ± 6.0	10.6 ± 4.2	12.7 ± 6.8	10.3 ± 4.8	11 ± 4.5	11.4 ± 8	4.7 ± 2.2
Calls handled rate	90%	98%	75%	94%	90%	91%	98%
Calls answered within 20 s (QS20)	62.5 ± 24.4	84.2 ± 16	36.3 ± 17.1	74 ± 16.4	47.1 ± 15.2	52.8 ± 17.4	80.7 ± 16.7
Occupation rate (%)	33.8 ± 13.9	29.6 ± 12.2	40.8 ± 14.7	29.2 ± 11.7	34.4 ± 13.8	34.4 ± 13.7	34.3 ± 13.5

The collection of activity data is based on a harmonised advanced telephony system in each EMCC.

### Selection of calls

In each centre, an advanced telephone system automatically keeps track of all inbound calls. According to the French national consortium, incoming calls that hung up in less than 15 s were considered as dialling errors and were excluded.

### Measurements and outcomes

The population data for each department were extracted from the National Institute of Statistics and Economic Studies website (https://www.insee.fr/fr/statistiques/1893198).

The advanced telephone system automatically keeps track of hourly activity, process measures and outcome measures. They were prospectively generated and stored within our advanced telephony system configured with CC Pulse + (Genesys, Alcatel Lucent, Daly City, California, USA).

We extracted some of these process measures and outcome measures, based on a previous study, for a one-year period beginning on 1 July 2016 [15]. We have selected these process measures and outcome measures as they are leverage in the operational analysis and are commonly used [9–11].

The primary outcome was the operational level of service, defined by the quality of service at 20 s (QS20). It corresponds to the rate of answered calls within 20 s. This is the quantitative quality requirement for assessing accessibility.

The secondary outcomes were “activity measures” (number of calls, call rate per 1000 inhabitants), “process measures” (incoming calls per agent, mean occupancy rate, average call duration). Those outcomes are defined in Table 1. The number of connected call takers was prospectively generated and stored within our advanced telephony system. A connected call taker could be busy on a call or available to handle an incoming call.

### Analysis

The results of this study are reported in accordance with the STROBE checklist for observational studies. The activity and performance indicators were described overall and by centre using the mean ± standard deviation. The activity of each centre was also analysed at different time granularities (whole study period, days of the week, hours of the day). We observed the curve of hourly variation in activity between centres.

We then described the correlations between the indicators using Spearman’s rank correlation. The estimated correlations were used to build SEM for the multivariate analysis [13]. These types of models allow estimating several regressions at once, as well as correlations between covariates. The estimations of the models are presented as standardised regression and correlation coefficients, which could vary between − 1 and 1. To estimate a potential centre effect, we compared an overall model with a multigroup SEM in which the coefficients were allowed to vary between centres. To allow comparability of these coefficients between centres, the intercepts of the regressions were kept equal. The goodness-of-fit of the models was estimated using the root mean square error of approximation (RMSEA), the comparative fit index (CFI) and the Tucker Lewis index (TLI). Good fitting models have values less than 0.08 for the RMSEA and greater than 0.95 for the CFI and TLI [16]. We analysed the data using R version 3.5.1 and fitted the models using the Lavaan package, version 0.5–20.

## Results

### General characteristics

Over the study period, we analysed 52,542 h of activity, during which 2,544,254 calls were received by the EMCCs, resulting in an overall annual call rate of 285.5 [95% CI: 285.2–285.8] per 1000 inhabitants. This rate ranged from 245.6 to 374.3 per 1000 inhabitants depending on the centre. The average number of call takers was 4.9 ± 1.5 connected operators per hour and varied from 4 to 6. Data per centre are presented in Table 2.

### Description of the activity

**Fig. 1:**
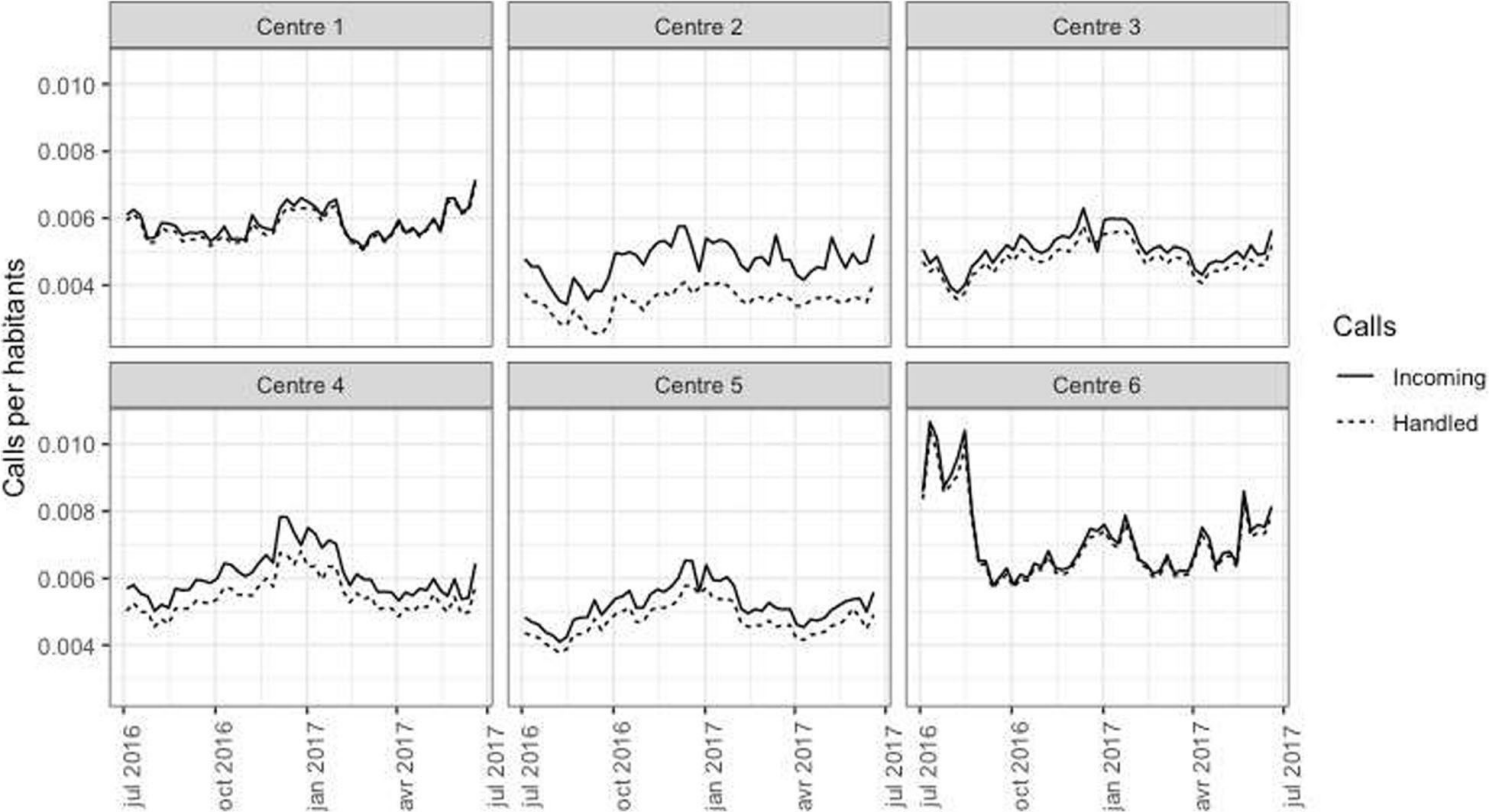
Median evolution of the number of incoming calls from the previous hour, by centre. Legend: A positive rate means an increase in the number of the incoming calls, and a negative rate means a decrease. At 9 AM, the evolution reached its highest level. The main increase occurred from 6 AM to noon

**Fig. 2:**
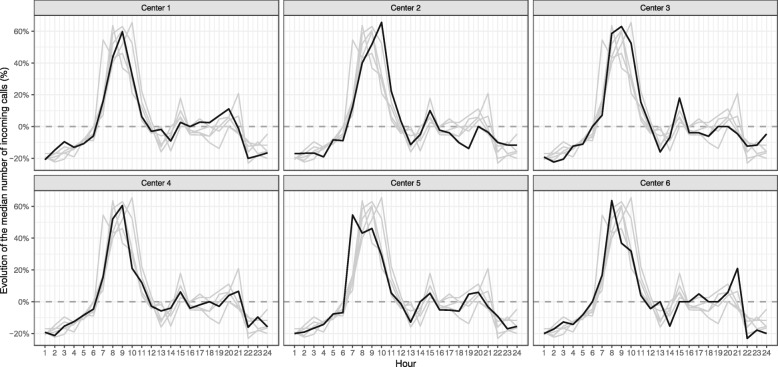
Results of the path analysis by structural equation models. Legend: Standardised coefficients estimated by centre from the structural equations models. Single-headed arrows represent regressions and double-headed arrows represent correlations between residuals. The standardised estimations are presented in bold for the initial model and between curly brackets for the multi-group model, as follows: {centre 1, centre 2, centre 3, centre 4, centre 5, centre 6}

Overall, the mean number of incoming calls and handled calls varied from 29.1 to 61.0 per hour and from 28.4 to 53.6 per hour, respectively. The evolution of each centre’s activity over the study period is presented in Fig. 1. Overall, call takers handled from 4.7 to 12.7 calls per hour, and the average call duration varied from 50.9 to 124.5 s. Within each centre, the number of incoming calls varied during the daycalls varied during the day (Fig. 2). An increase in the number of incoming calls generally started 6 AM and continued until noon, but with varying slopes depending the centre. From 4 PM to 9 PM, the variation in the number of incoming calls was different between centres.

### Accessibility, activity measures and process measures

Over the study period, the proportion of calls handled ranged from 75 to 98%. The average proportion of calls handled within 20 s (QS20) varied from 36.7 to 84.2%. The occupation rate ranged from 29.2 to 40.8%. Results per centre are presented in Table 2.

### Structural equation modelling

**Fig. 3:**
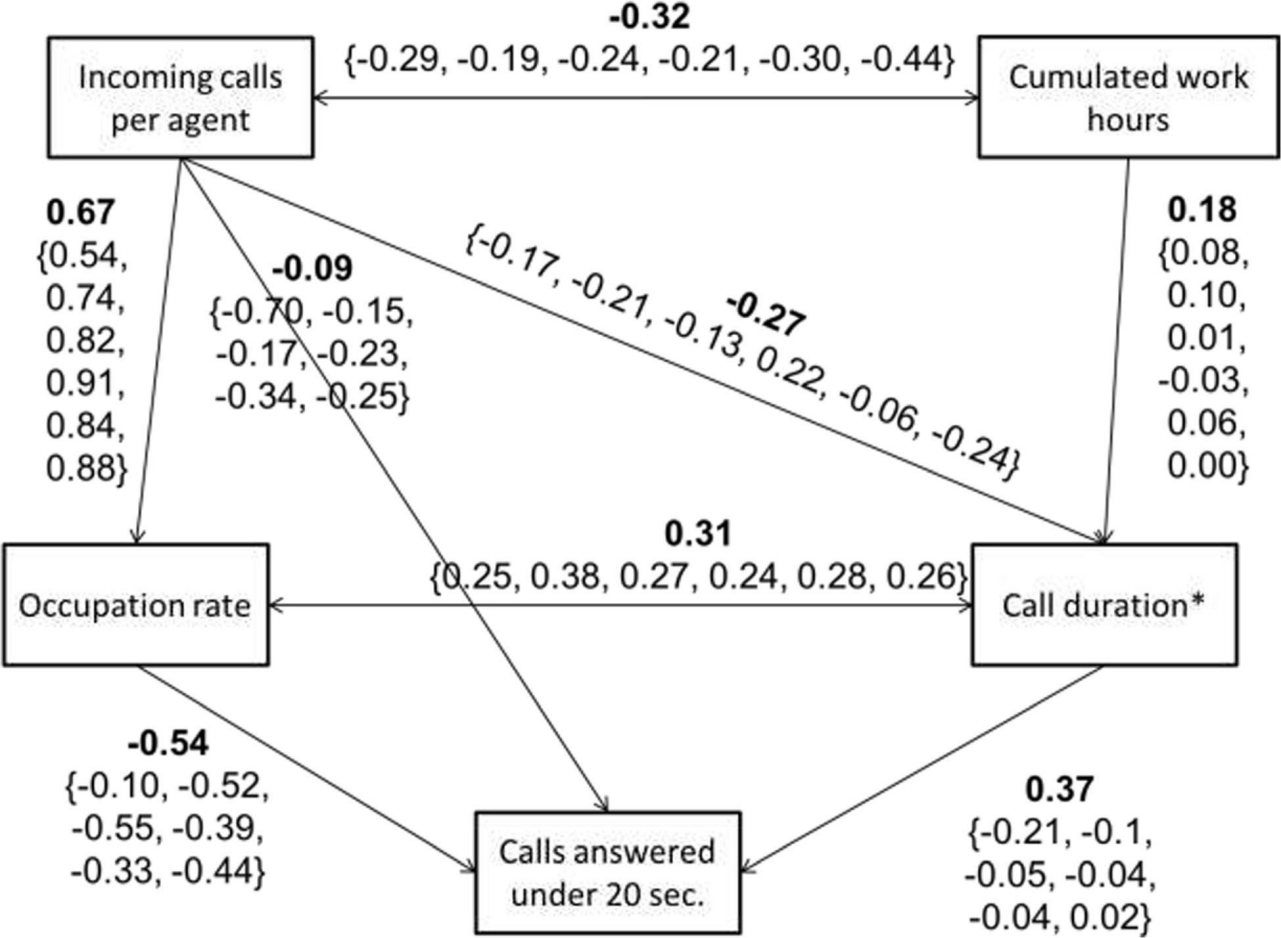
Numbers of incoming and handled calls per inhabitant over a year, by centre. Legend: Data are aggregated by week. Incoming calls are represented with full lines and the handled calls with dotted lines

The results of the models are presented in Fig. 3. All the indicators showed a good fit to the data of the overall model (TLI = 0.997, CFI = 0.999, RMSEA = 0.024). However, it was significantly improved by letting the coefficients vary between centres (*p* < 0.001). The resulting multi-group SEM also exhibited good fit indices (TLI = 0.990, CFI = 0.994, RMSEA = 0.047).

In both models, the largest effects were observed between the number of incoming calls per agent and the mean occupation rate (0.54 to 0.91) and between the mean occupation rate and the QS20 indicator (− 0.10 to − 0.55). A large direct effect was also observed between the number of incoming calls per agent and the QS20 indicator, but only for centre 1 (0.70). The addition of the centre effect via the multi-group model also modified the size of some effects: while the effect of the call duration on the QS20 indicator was equal to 0.37 in the initial model, it was smaller in the multi-group model (− 0.21 to 0.02). The same phenomenon can be observed for the effect of the mean cumulated hours of work and the log average call duration.

## Discussion

### Main results

Our study represents the largest series of emergency calls analysed in a multicentre analysis in the area of population accessibility to EMCCs. The workload increased the agent occupancy rate and degraded the QS20. A saturation effect exists in call taker productivity, and this limits the population access to EMCCs. Temporal variation in EMCC activity was different between the centres more than a third of the daily time. Our work illustrates that activity and process measures are not homogeneous between centres even if a correlation exists.

### Explanation of the findings

In the call centre literature, quite a few studies have evaluated the quality and performance of EMCCs. These articles are mainly about methods for improving the triage and dispatching processes [7, 17, 18]. To the best of our knowledge, less is known about the accessibility of EMCCs, and improvement will require knowledge of activity and process measures to optimise the scheduling process. Improving accessibility, managing workload and performing triage are converging objectives. Indeed, depending on the time of the call and the workload, the categorisation of the call can be difficult and have an impact in terms of mortality for patients [19]. The main effects in our models to explain the variations in the QS20 indicators were observed for indicators of the workload of the operators: the number of incoming calls per call taker had the largest impact on the mean occupation rate, which had the largest impact on QS20. These results are consistent with the results of a study that we previously conducted in one of the centres, showing that the main lever identified to improve the quality of service in EMCCs is to reduce the occupation rate of the operators [15]. The other main finding of our study is the importance of the heterogeneity observed between centres. Indeed, the estimations of the models varied significantly between centres according to the multi-group model. As seen by the differences between the overall and multi-group models, the centre was a confounding factor, and it is probable that organisational factors that vary between centres can explain the correlation between call durations and QS20, without a direct link between the two. Efforts to reduce the average call duration will only have limited effects on the QS20 according to our multi-group model. On the other hand, Raknes et al. considered the reduction of the average call duration as a measure to increase EMCC capacity [9]. We noticed a saturation effect in call taker productivity. Although centres 3, 4 and 5 have a lower average call duration, agent productivity is not improved. Indeed, the mean number of calls handled hourly per call taker is rather comparable in centres 1 to 5, even if the mean call duration varied from 50 s to more than 100 s. This might be the sign of a saturation effect: below a certain point, decreasing the duration of the calls will have no effect on the productivity and quality of service. This hypothesis is also raised by the proportionality between the number of handled calls and the number of incoming calls illustrated in Fig. 1. In centres 1 and 6, the curves are superimposed. In centres 2 to 5 they are not, but the difference seems to be constant, confirming the potential saturation effect. We hypothesised that centre 2 seems to be understaffed throughout the year and centre 6 has a different curve due to a touristic seasonal effect.

The annual EMCC call rate was 285.5 per 1000 inhabitants. This was consistent with the emergency department call rate [20]. This is one of the first studies describing this hourly variability. Previous studies described the daily activity [15, 21]. Moller et al. described the diurnal variation of incoming calls and our results are consistent [22]. Variations in activity between EMCCs are observed from 4 PM to 9 PM. This illustrates that EMCCs differ in terms of temporal activity. Several factors could explain these variations in activity as differences in geographical location of the EMCCs, population movements (urban area, rural area) or local health organisation. For example, the centre 6 receives calls from a mixed population (rural and urban). The proportion of inhabitants over 75 years old is the highest among the 6 centres in the study, according to the National Institute of Statistics and Economic Studies website (https://www.insee.fr/fr/statistiques/2012692). Even if the average socio-economic level is good, there are inequalities that can explain a higher call rate [23]. The center 2 is an urban center with a high population density. The abandonment rate is probably related to a shortage of call-takers and the possibility of calling another emergency number in the event of an immediate non-response. The fire brigade, with another telephone number, is available nation-wild. Home visit general practitioners services are numerous but their distribution on the territory is very variable. There are particularly represented in the area of the center 2.

### Limitations

One limitation of our study is precisely that we lacked data to identify the causes of local variations. According to statistical process control, process indicators such as the ones we used in this study can vary because of two types of phenomena called common causes and special causes. Common causes are random variability due to the process in itself, such as the expected variability between calls, call takers, etc. Special causes have a clear identifiable cause, such as a rise in activity due to a particular event or lower performance indicators after a change in the telephone system. These two types of causes of heterogeneity should be explored in future studies to better predict EMCCs’ performance and quality of service. Another limitation is the fact that we used a convenience sample rather than an exhaustive or random sample. We used this methodology in the aim to have harmonized data despite the risk of selection bias. In order to be nationally representative, it would be necessary to sample the EMCCs on the basis of a balance of type of population covered (rural, mixed, urban) and population size or to conduct a larger study. France is in the process of harmonising its regulation software, which will allow this big data analysis to be carried out. However, it seems improbable that including other centres would have reduced the heterogeneity that we observed in our study, and our main conclusion would therefore not have changed.

## Conclusions

The agent occupancy rate is negatively correlated with and decreases the QS20. The scheduling process must consider this negative impact in order to optimise the EMCCs’ response time. There is individual EMCC variability in the temporal distribution of inbound calls and in the analysis of activity and process measures due to organisational factors that will need to be studied.

## Data Availability

The database, statistical code and technical processes are available from the time of publication. Requests should be made via email to the corresponding author along with an analysis proposal.

## Abbreviations

CFI: Comparative fit index
EMCC: Emergency Medical Communication Centre
QS20: Quality of service at 20 s
RMSEA: Root mean square error of approximation
SAMU: Service d’aide médicale urgente
SEM: Structural equation models
TLI: Tucker Lewis index
